# Variations in Blood Platelet Proteome and Transcriptome Revealed Altered Expression of Transgelin-2 in Acute Coronary Syndrome Patients

**DOI:** 10.3390/ijms23116340

**Published:** 2022-06-06

**Authors:** Rafał Szelenberger, Paweł Jóźwiak, Michał Kacprzak, Michał Bijak, Marzenna Zielińska, Alina Olender, Joanna Saluk-Bijak

**Affiliations:** 1 Department of General Biochemistry, Faculty of Biology and Environmental Protection, University of Lodz, 90-236 Lodz, Poland; joanna.saluk@biol.uni.lodz.pl; 2 Biohazard Prevention Centre, Faculty of Biology and Environmental Protection, University of Lodz, 90-236 Lodz, Poland; michal.bijak@biol.uni.lodz.pl; 3 Department of Cytobiochemistry, Faculty of Biology and Environmental Protection, University of Lodz, 90-236 Lodz, Poland; pawel.jozwiak@biol.uni.lodz.pl; 4 Department of Interventional Cardiology, Medical University of Lodz, 91-213 Lodz, Poland; michal.kacprzak@umed.lodz.pl (M.K.); marzenna.zielinska@umed.lodz.pl (M.Z.); 5 Chair and Department of Medical Microbiology, Medical University of Lublin, 20-093 Lublin, Poland; alinaolender@umlub.pl

**Keywords:** blood platelets, proteome, transcriptome, acute coronary syndrome, transgelin-2

## Abstract

Proteomic analyses based on mass spectrometry provide a powerful tool for the simultaneous identification of proteins and their signatures. Disorders detection at the molecular level delivers an immense impact for a better understanding of the pathogenesis and etiology of various diseases. Acute coronary syndrome (ACS) refers to a group of heart diseases generally associated with rupture of an atherosclerotic plaque and partial or complete thrombotic obstruction of the blood flow in the infarct-related coronary artery. The essential role in the pathogenesis of ACS is related to the abnormal, pathological activation of blood platelets. The multifactorial and complex character of ACS indicates the need to explain the molecular mechanisms responsible for thrombosis. In our study, we performed screening and comparative analysis of platelet proteome from ACS patients and healthy donors. Two-dimensional fluorescence difference gel electrophoresis and nanoscale liquid chromatography coupled to tandem mass spectrometry showed altered expressions of six proteins (i.e., vinculin, transgelin-2, fibrinogen β and γ chains, apolipoprotein a1, and tubulin β), with the overlapping increased expression at the mRNA level for transgelin-2. Dysregulation in protein expression identified in our study may be associated with an increased risk of thrombotic events, correlated with a higher aggregability of blood platelets and induced shape change, thus explaining the phenomenon of the hyperreactivity of blood platelets in ACS.

## 1. Introduction

Mass spectrometry-based proteomic analysis constitutes a powerful tool that allows parallel identification of proteins from the cells, tissues, and biological fluids. Current applications cover and facilitate the evaluation of alterations in protein expression, structure, and function. A wide range of applications allows for assessing specific protein signatures, novel biomarkers for disease diagnosis, and progression evaluation as well as new drug targets. Modern prognostic diagnostics depend on finding the biochemical, cellular, or genetic alteration by which pathological conditions in the human body can be recognized early and prevent the disease state from occurring. Recent advances in molecular techniques, technologies, and bioinformatics opened new possibilities to study genomics, proteomics, metabolomics, and imaging. These techniques have a tremendous bearing on having a better understanding of disrupted cellular mechanisms during an ongoing pathological state. The etiology of the disease has a great impact on the selection of proper methodology, which may offer better prognostic, diagnostic, and therapeutic value [1,2].

According to the data presented by the World Health Organization (WHO), acute coronary syndrome (ACS) and stroke constitute 85% of all cardiovascular diseases and are the most common causes of death globally, taking 17.9 million lives each year. Although most cardiovascular diseases can be prevented through appropriate behavior and lifestyle and high morbidity and mortality rates emphasize their variable, unpredictable, nonlinear, and clinically silent character [3].

One of the key roles in the thrombosis and pathogenesis of ACS is played by blood platelets, which are the smallest and anucleate morphotic elements. Under physiological conditions, platelets freely circulate in the bloodstream without stimulating pivotal interactions with the vascular microenvironment. However, in the result of vessel injury, platelets induce the hemostasis process which includes complex interplay among all elements of the circulatory system [4]. In the first stage of primary hemostasis, platelets adhere to the damaged vessel wall and interact with the subendothelial matrix proteins. To ensure the stable contact between platelets and the injured endothelium, via surface receptors, platelets bind with the collagen and von Willebrand Factor (vWF). Initial binding is vouched by the glycoprotein (GP) Ib-IX-V, which recruits blood platelets from circulation, reduces their velocity, and allows them to induce the interaction in multiple directions (i.e., by stimulating interaction with leukocytes, endothelium, and other platelets). Further, platelets bind with damaged endothelium by other surface receptors, including GP VI and GP Ia/IIa, thus enhancing the stability of the mutual connection and initiating platelet activation [5]. The signal transduction from the agonists released by platelets causes terrific changes in their physiology. Stimulated platelets change their shape, form numerous pseudopods, expose more receptors on their surface, and release from the α- and dense granules a vast amount of biologically active molecules that enhance their activation, aggregation, and stimulate the development of inflammation [4]. An ongoing process of platelet activation promotes the conformational changes in the structure of the most potent platelet receptor—GP IIb/IIIa, which causes the formation of fibrinogen bridges between activated platelets. During the aggregation process, the platelet response is continuously amplified to induce the growth of the forming thrombus. The multifactorial hemostatic response caused by excessive activation of blood platelets, in the final result induces the occlusion of the vessels and limits the blood flow, causing thrombotic complications such as ACS [6]. ACS patients were shown to present persistent platelet hyperreactivity [7], which may be strongly associated with the pathological formation of blood clots in coronary arteries. Thus, a better understanding of the potential alterations on the molecular level may be crucial for a faster inclusion of preventive approaches and/or assessing the potential risk of ACS development that could reduce its morbidity and mortality.

Because of the complex, multifactorial character of ACS, the search for potentially important alterations should be performed at various levels to identify possible interactions or mechanisms associated with the ischemia. The role of blood platelets in the pathogenesis of thrombosis is well established; however, their unique properties (i.e., lack of nucleus and transcription process, which significantly limits the pool of presented proteins; easy, non-invasive method to collect samples) show that platelet proteome could be an ideal area for the discovering new targets that could be linked with the ACS development. The main objective of our study was to find and identify, through the proteomic comparative analysis, dysregulated platelet proteins along with their expression at the mRNA level that could provide new insights into the molecular mechanism responsible for hyperreactivity and spontaneous activation of platelets associated with ACS.

## 2. Results

### 2.1. Analysis of Fluorescence Intensity in Two-Dimensional Fluorescence Difference Gel Electrophoresis

**Figure 1:**
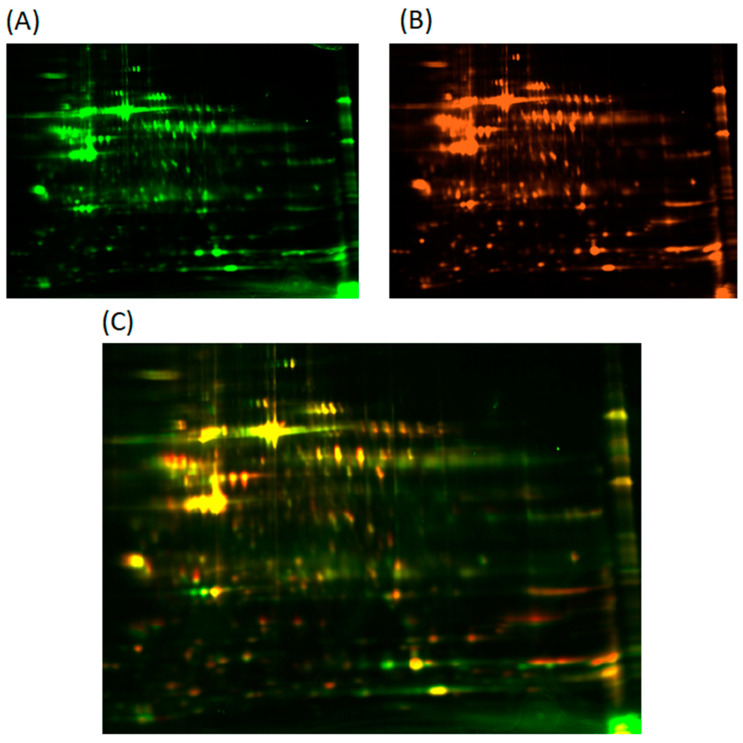
Fluorescently labeled electrophoretic gels of blood platelets proteome obtained from the control group (**A**) and ACS patients (**B**). Merge spots (**C**) represent the comparison between proteomes.

To determine the differences in platelet proteome between patients diagnosed with ACS and the control group, clinical material was first analyzed using two-dimensional fluorescence difference gel electrophoresis (2D-DIGE). To avoid any unexpected mechanical and technical problems, two gels were prepared simultaneously as a separate analysis. Scanned gels for both groups are presented in Figure 1. For each spot, the range ratio value, which corresponded to the differences in fluorescent intensity between the study and control group, was assessed. For spots S1–S6, the range ratios were 3.05223, 1.72152, 1.99851, 2.38866, 2.12995, and 1.94427, respectively. Furthermore, spots S1 and S4 were downregulated, while other spots were upregulated.

### 2.2. Identification of Altered Expressed Proteins via Nanoscale Liquid Chromatography Coupled to Tandem Mass Spectrometry

**Figure 2:**
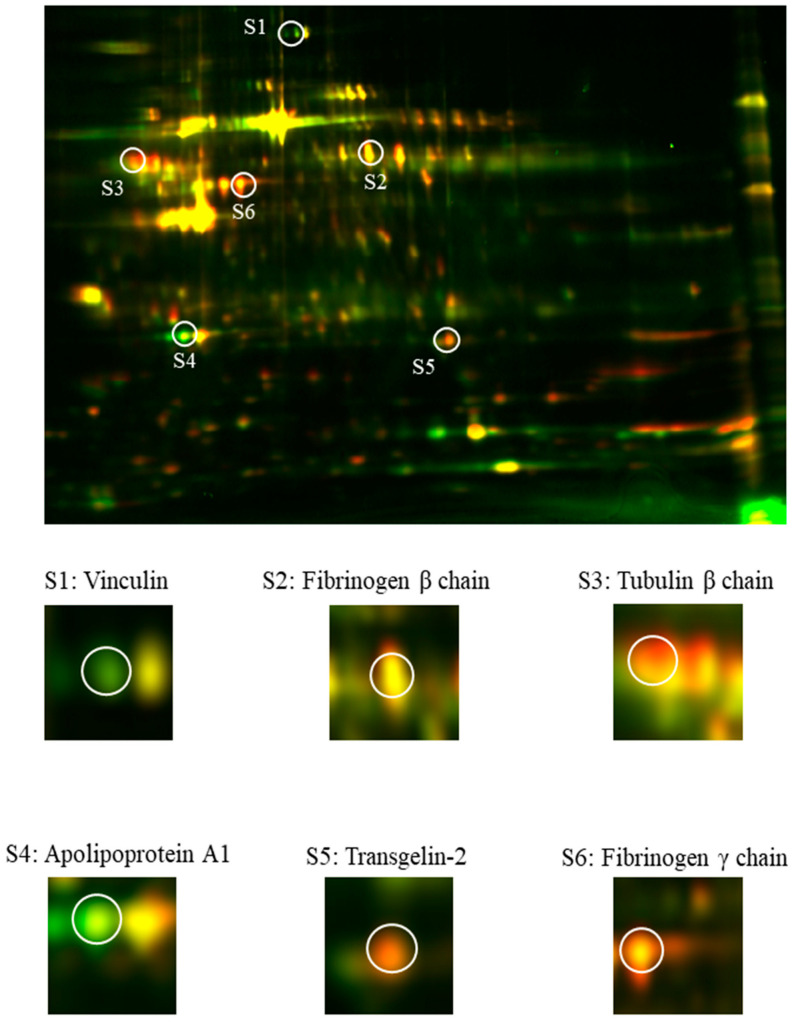
Identification of selected protein spots from blood platelets of ACS patients via nanoscale liquid chromatography coupled to tandem mass spectrometry.

To identify the protein features, nanoscale liquid chromatography coupled to tandem mass spectrometry (nanoLC-MS/MS) was carried out for six (S1–S6) chosen spots. Differentially expressed proteins were divided into four functional groups: actin-binding proteins (S1: vinculin; S5: transgelin-2), cytoskeleton (S3: tubulin β chain), lipid metabolism (S4: apolipoprotein a1), and blood clot formation (S2: fibrinogen β chain; S6: fibrinogen γ chain). Altered expressed proteins are visualized in Figure 2 and listed in Table 1.

### 2.3. mRNAs Expression Levels in Blood Platelets

**Figure 3:**
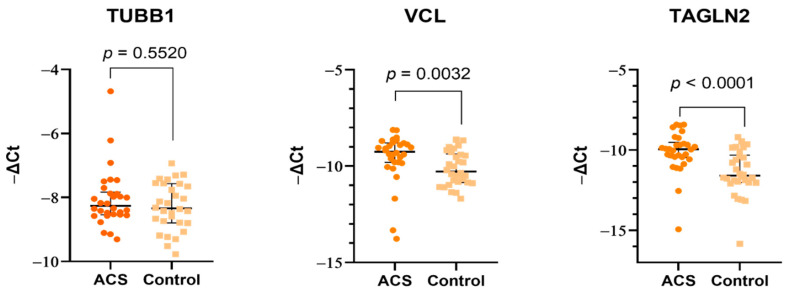
The mRNA expression levels of TUBB1, VCL, and TAGLN2 in blood platelets of ACS patients and healthy donors. The relative expression was assessed by the −ΔCt value. As a reference gene, 18S rRNA was used. Data are plotted as individual values with horizontal bars representing medians and interquartile range. *p*-Values were calculated using the Mann–Whitney U test.

Our comparative analysis showed that of the six analyzed genes encoding apolipoprotein A1 (*APOA1*), vinculin (*VCL*), transgelin-2 (*TAGLN2*), tubulin β-1 chain (*TUBB1*), fibrinogen β chain (*FGB*), and fibrinogen γ chain (*FGG*), three did not show expression in blood platelets (i.e., APOA1, FGB, and FGG), two were significantly upregulated in ACS patients (i.e., VCL and TAGLN2), and one did not differ between studied groups (i.e., TUBB1) (Figure 3). The lack of expression was associated with no observed formation of product and did not exceed the threshold during analysis.

### 2.4. The Concentration of Fibrinogen in Plasma

**Figure 4:**
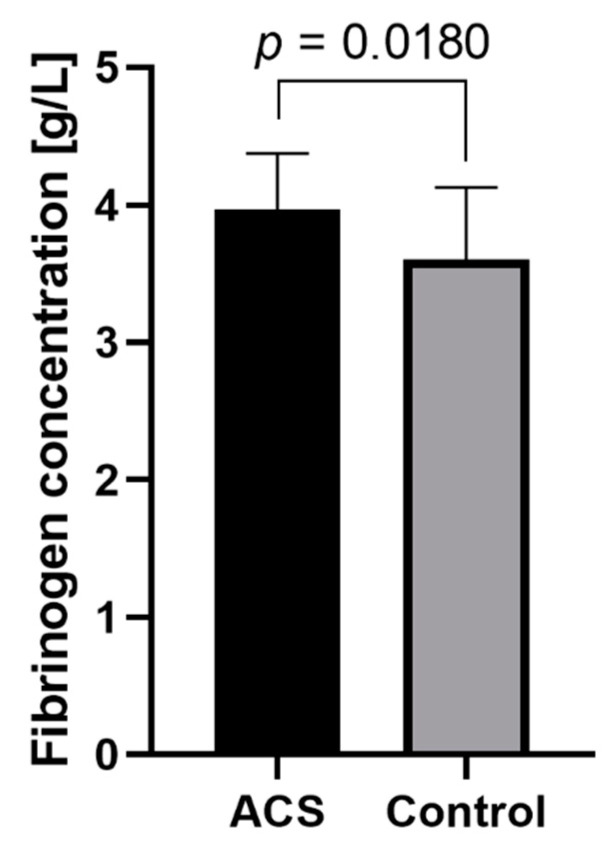
The comparison of plasma fibrinogen levels in ACS and control groups. Data presented in the graph show the mean concentration ± SD. Obtained results passed the Shapiro–Wilk’s test, and the unpaired Student’s *t*-test was used to calculate the differences.

To verify that the augmented level of fibrinogen is exclusive to ACS blood platelets, the measurement of fibrinogen concentration was performed on the plasma samples. Obtained results from the enzyme-linked immunosorbent assay (ELISA) experiment showed that in the plasma of ACS diagnosed patients, the fibrinogen level was significantly increased in comparison to the control group: 3.968 ± 0.404 g/L in the study group vs. 3.601 ± 0.525 g/L in the control group (*p* = 0.018) (Figure 4).

### 2.5. Validation of Transgelin-2 Concentration in Blood Platelets by Western Blot

**Figure 5:**
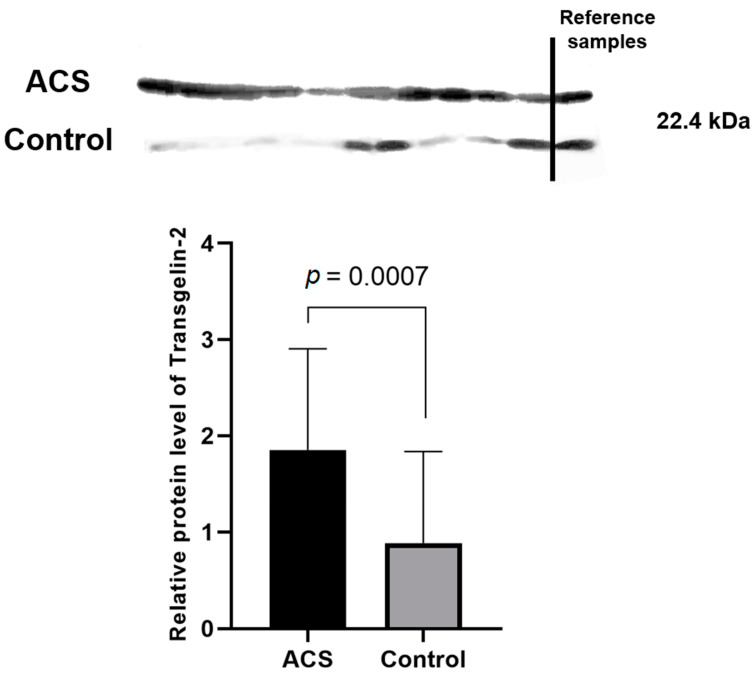
Expression of transgelin-2 protein in blood platelets of ACS patients and control subjects. The integrated optical density (IOD) was analyzed by densitometry and normalized by the protein concentration and reference sample. The graph demonstrates the median value from IODs with an interquartile range (25–75%). Data were calculated using the Mann–Whitney U test.

Densitometric analysis of the bands corresponding to the transgelin-2 from blood platelets showed statistically significant augmented expression in ACS patients with a median of 1.851 (1.286–2.903) compared to healthy volunteers with a median of 0.885 (0.215–1.835) (*p* = 0.0007; range ratio = 2.091) (Figure 5).

## 3. Discussion

The pathophysiology of coronary arteries thrombosis is complex and includes not only the classic risk factor, such as age, gender, dyslipidemia, arterial hypertension, diabetes, lack of physical activity, or smoking, but also an interplay between many genes that by regulating biochemical pathways, modify the risk of developing arterial thrombosis [8]. The role of blood platelets in thrombosis is well known [9]; however, the phenomenon of hyperreactivity of platelets and its undesirable interactions with other types of cells may lead to various, life-threatening complications including ACS. The participation of blood platelets in vasoconstriction, microembolization, and atherosclerotic plaque progression contributes to inherent persistent platelet hyperreactivity, resulting in the augmented risk of ischemic events [7,10]. The main goal of our study was to find and identify overlapping alterations in the proteome and transcriptome of blood platelets that could help in a better understanding of the molecular mechanisms of thrombosis, platelet behavior, and contributes to the development of new approaches for evaluating the risk of ACS occurring.

Our comparative analysis showed the presence of six differentially expressed proteins (i.e., vinculin, tubulin β chain, fibrinogen β and γ chains, apolipoprotein A1, and transgelin-2) in ACS patients in comparison to donors without any cardiovascular system disturbances. Vinculin is a membrane-cytoskeletal protein that serves as a linker between membrane-associated proteins and the actin cytoskeleton, thus regulating cell adhesion and cell–cell interactions [11]. To evaluate the role of vinculin in blood platelets, Mitsios et al. performed a study in which platelet functions were studied in vinculin knockout mice. Surprisingly, the results showed that vinculin-deficient platelets did not reveal any disturbances and functioned normally [12]. Our 2D-DIGE analysis showed an over three-fold decrease in the concentration of vinculin (Table 1). A similar result was obtained by Lopez-Ferre et al. in a study in which proteomic analysis of ACS patients showed a significantly downregulated expression of vinculin [13]. Although the concentration of vinculin in our study was reduced, the expression on the mRNA level of VCL was significantly augmented. A possible explanation may be associated with the post-transcriptional modifications and the role of microRNA (miR). In our previously published study [14], we showed that blood platelet possesses an elevated level of hsa-miR-21-5p, which was shown to target VCL transcripts [15]. The various directions of changes in proteome and transcriptome, and the lack of a direct effect for platelets in the mice model suggest that the role of vinculin may not be crucial for thrombosis. Moreover, the decreased concentration of vinculin should be associated with the lower platelet response to adhesion and cell–cell interactions, which seems to be contrary to the nature of ACS pathogenesis.

The major components of the platelet cytoskeleton are microtubules, which play an essential role in maintaining cell shape and its changes and participating in intracellular transport. The most important components of microtubules are two isoforms of tubulin protein: α-tubulin, the specific role of which in blood platelets is not fully understood, and β tubulin, which is more abundant and essential for microtubule and platelet formation. Their movement from the center to the cell cortex initiates the creation of pseudopods, which have elongations that lead to the formation of proplatelets. Furthermore, microtubules are essential for maintaining the discoid and/or spherical shape of blood platelets [16]. A study performed on β1-tubulin-deficient mice showed a prolonged bleeding time, weaker response to thrombin, and a lack of discoid shape [17], thus supporting the important role of microtubules in platelet function. Unfortunately, there are no studies concerning the overexpression of tubulins and their potential role in the dysregulation of platelet function. The increased concentration of tubulin β chain (Table 1) was not reflected at the mRNA level (Figure 3), which suggests that molecular alterations may arise directly in megakaryocytes. However, it requires further detailed research focus not only on platelets but also on their precursor cells.

Preventing blood loss by ensuring the continuity of the blood vessel following vascular injury is the most important process in hemostasis. The activation of the coagulation cascade and blood platelets leads to the enzymatic conversion of fibrinogen to insoluble fibrin, the main component of blood clots. Fibrinogen is a homodimeric glycoprotein of an acute phase, composed of two sets of three different polypeptide chains (i.e., Aα, Bβ, and Yγ) linked by 29 disulfide bridges. Fibrin deposition tethers the circulating cells enhancing the primary platelet plug and extending a fibrin network, eventually forming a clot, preventing blood extravasation or in pathological conditions leading to a significant reduction in the vascular lumen or even complete vessel occlusion [18]. Studies have showed that elevated levels of plasma fibrinogen are associated with an increased risk of thrombotic events in patients and correlated with higher aggregability of blood platelets [19,20,21]. In 2015, Appiah et al. performed a large prospective study in which the level of the plasma fibrinogen γ chain was positively associated with the occurrence of coronary artery disease, ischemic stroke, heart failure, peripheral artery disease, and even death (caused by cardiovascular diseases) [22]. Data combined from 53 prospective studies showed that evaluations of fibrinogen levels in male patients with an intermediate risk of cardiovascular diseases could significantly increase the efficiency of predicting the disease and help to prevent one event for every 400–500 people screened over 10 years [23]. Our comparative analysis showed a statistically significant upregulation in spots identified as either a fibrinogen β and γ chain in blood platelets samples from ACS patients (Table 1). Fibrinogen is synthesized in the liver and released into the bloodstream in concentrations ranging from 1.5 to 4 g/L [24]. Due to the assessment of the possible molecular disorders associated with the ability of platelets to synthesize the fibrinogen, the levels of mRNA transcripts for FGB and FGG were evaluated. The results showed that blood platelets lacked fibrinogen transcripts, which suggests that its intraplatelet augmented level is not associated with possible synthesis in platelets. Further, the level of plasma fibrinogen was assessed between the studied groups. Received data showed a statistically significant increase in plasma fibrinogen concentration in patients with ACS (Figure 4). Despite the differences between the studied groups, the mean values fit within the physiological range of fibrinogen concentration. Unfortunately, the obtained results enable to pinpoint the direct cause of the increased fibrinogen levels. The overexpression in the 2D-DIGE analysis may be caused by the presence of plasma fibrinogen residues, as platelets were isolated from plasma. On contrary, the elevated concentration of plasma fibrinogen may be linked with the process of releasing the platelet granule content in the response to their activation. The increased reactivity of blood platelets may also be associated with the elevated level of fibrinogen receptor, GP IIb/IIIa, on the blood platelets’ surface [7]. Augmented exposition of GP IIb/IIIa to the higher fibrinogen level could result in the effective, stronger, and more willing activation of blood platelets in ACS patients. Due to the lack of the possibility of a precise explanation of the obtained results, further detailed studies are necessary to determine the fibrinogen level without the potential background from both sides: platelets and plasma. Especially, because of platelets’ ability to take up proteins from plasma or other cells [25].

Apolipoprotein A1 is a crucial protein that constitutes a functional and structural component of high-density lipoprotein (HDL) [26]. Studies showed that HDL and apolipoprotein A1 possess an ability to block vWF self-association resulting in the formation of shorter fibers. In patients with hyperadhesive vWF disorders, apolipoprotein A1 was significantly reduced in comparison to healthy controls, thus suggesting the vWF-specific antithrombotic property of apolipoprotein A1 and HDL. The antithrombotic capacity of HDL-apolipoprotein A1, however, affected platelet adhesion only to the reduced formation of vWF fibers, thus suggesting that they are not influencing the platelet activity directly [27]. In our study, HDL and apolipoprotein A1 levels were significantly decreased in ACS patients (Table 1 and Table 2) which, according to Chung et al.’s study, may be implicated with the decreased level of antithrombotic and antiadhesive properties, thus increasing risk of thrombosis. A similar result was obtained in the Cevik et al. study, in which apolipoprotein A1 was downregulated in acute ischemic stroke patients [28].

The most interesting and important finding in our study was the significant elevation in the proteomic and transcriptomic levels of Transgelin-2. Transgelin-2 is a small ~22 kDa protein that belongs to the actin-associated protein group and is one of three known transgelin isoforms. The specific function of transgelin-2 in platelets has not yet been determined; however, available studies have showed that the principle role of transgelin-2 is linked with actin polymerization [29], which in blood platelets plays a very important role in shape change, morphogenesis, migration, and apoptosis [30,31]. Moreover, data from the UniProt and GeneCards databases indicate that transgelin-2 can be involved in processes of platelet degranulation, platelet activation, signaling, aggregation, and response to elevated platelet cytosolic Ca^2+^ levels [32,33]. The C-terminal calponin-like repeated region and the N-terminal single calponin-homolog domain, which is a potential Ca^2+^ binding site, can be distinguished in the structure of transgelins. Furthermore, transgelins can also act as suppressors for the expression of metallo-matrix proteinase 9 (MMP-9) [34]. In a study conducted by Sheu et al., activated MMP-9 significantly reduced platelet aggregation induced by thrombin, arachidonic acid, adenosine diphosphate, U46619, and collagen, suggesting that this phenomenon may be associated with the inhibition of Ca^2+^ mobilization [35]. The suppression of MMP-9 may affect blood platelets to exhibit a poorer mechanism of natural inhibition, which may result in their hyperreactivity. Furthermore, the ability of transgelin-2 to bind F- and G-actin resulted in specific localization in the actin structures such as podosomes or stress fibers [36]. It was suggested by Poulter et al. that platelet podosome-related structures may be crucial for the platelet–platelet interactions [37]. The results of our study showed for the first time the altered expression of transgelin-2 in blood platelets on the proteome (Table 1, Figure 5) and transcriptome levels (Figure 3). The 2D-DIGE and Western blot analyses showed an over two-fold increase in the concentration of transgelin-2 in the blood platelets of ACS patients in comparison to healthy donors (*p* = 0.0007). The expression at the mRNA level also significantly increased in the study group. We speculate that the increased amount of transgelin-2 may be associated with the stimulation of changes in the shape of blood platelets by regulating actin polymerization and the formation of structures, such as lamellipodia or pseudopodia, which may contribute to a spontaneous reaction of platelet adhesion, activation, aggregation, and/or translocation of receptors responsible for interactions with other types of cells.

Similar studies have been carried out over the last 10 years; however, different proteins were identified [14,28,38,39,40]. Our study had a big advantage due to the use of fluorescent labeling, which shows the most sensitive detection and is the most suitable for use with mass spectrometry. Moreover, the usage of only one gel limits the need for replicates because of the co-migration of two different protein samples as a single spot [41]. In addition to a few overlapping results, our study provides new insights into the molecular changes in the proteome and transcriptome of blood platelets. For the first time, we showed an altered expression of transgelin-2, which may contribute to the processes responsible for the degranulation and shape change of platelets. The role of transgelin-2 in platelets remains unclear; however, it seems to have a potential impact on thrombosis and may partially explain the molecular disorders responsible for the hyperreactivity of blood platelets. Finding new measurable indicators of pathological, unwanted activation of blood platelets may help in identifying new targets for future therapy, monitoring the medical condition, and in assessing the probability of the future incidence of ACS in potential patients.

## 4. Materials and Methods

### 4.1. Chemicals

KCl, glucose, urea, thiourea, 2-(4-(2-hydroxyethyl)piperazin-1-yl)ethanesulfonic acid (HEPES), 3-((3-cholamidopropyl)dimethylammonio)-1-propanesulfonate (CHAPS), Tris, bovine serum albumin (BSA), K_3_[Fe(Cn)_6_], iodoacetamide, trypsin from bovine pancreas (sequencing grade), and NH_4_HCO_3_ were purchased from Sigma-Aldrich (St. Louis, MO, USA). NaCl, NaH_2_PO_4_, NaHCO_3_, trifluoroacetic acid optima (TFA), and citrate were purchased from POCh (Gliwice, Poland). Na_2_SO_3_ was purchased from Alfa Aesar (Haverhill, MA, USA). Acetonitrile (AcN) LC-MS was purchased from Chempur (Piekary Slaskie, Poland). Dithiothreitol (DTT) was purchased from GE Healthcare (Chicago, IL, USA). PBS tablets were purchased from Biosigma (Venice, Italy).

### 4.2. Characteristics of Patients and Donors

Blood samples were drawn from 30 patients admitted to the Department of Interventional Cardiology of the Medical University of Lodz via the S-Monovette system (Sarstedt, Numbrecht, Germany) with CPDA-1 anticoagulant. The ACS incident was confirmed angiographically. The inclusion and exclusion criteria were the same as previously described [14,42]. The control group was homologous to the study group in terms of number, age, and sex. Blood samples from the control group were collected by BD Vacutainer^®^ (Becton Dickinson, Franklin Lakes, NJ, USA)probes with ACD-A as an anticoagulant in the Laboratory Diagnostics Centre in Lodz. All donors qualified for the study were subjected to the following blood tests: morphology, creatinine, coagulation, TSH (thyroid-stimulating hormone), IgG and IgM titers (immunoglobulin G and M), CRP (C-reactive protein), AST (aspartate transaminase), ALT (alanine transaminase), triglycerides, total cholesterol, HDL, LDL (low-density lipoprotein), and glucose. All registered participants in the control group did not administer any medications for at least 2 weeks before blood collection and were free from any illness. Furthermore, patients and donors were not previously diagnosed with any cardiovascular diseases. The study was approved by the Committee of Ethics of Research in Human Experimentation at the University of Lodz with resolution number 23/KBNN-UŁ/I/2017. Patients and volunteers enrolled in the study signed an informed consent form before inclusion. All procedures were carried out according to the Helsinki Declaration for Human Research. Clinical characteristic of patients and donors is included in Table 2.

**Table 2:** Characteristics of ACS patients and healthy donors.

Parameter	ACS (*n* = 30)	Control (*n* = 30)	Reference Range	*p*-Value
	Median (1st–3rd Quartiles) or Number (Frequency)			
Age (years)	50 (45–61)	49 (41–57)	-	0.594
Sex (male)	25	25	-	-
BMI (kg/m^2^)	30 (26–31)	29 (26–32)	<35	0.874
Leukocytes (10^3^/µL)	8.42 (7.00–9.65)	6.00 (4.83–7.73)	4–11	<0.001
Erythrocytes (10^6^/µL)	4.42 (4.18–4.95)	5.03 (4.53–5.33)	4.2–6.1	0.011
Blood platelets (10^3^/µL)	246 (200–281)	251 (217–300)	150–400	0.478
Glucose (mmol/L)	6.00 (5.31–6.28)	4.96 (4.73–5.46)	4.1–5.5	<0.001
Creatinine (µmol/L)	81.0 (71.7–88.7)	75.6 (69.6–86.8)	64–104	0.291
GFR (ml/min/1.73 m^2^)	96.8 (80.3–104.3)	93.4 (81.6–104.2)	>60	0.921
AST (U/I)	34 (25–38)	19 (16–24)	<50	<0.001
ALT (U/I)	28 (19–39)	20 (14–34)	<50	0.116
Total cholesterol (mmol/L)	4.90 (4.21–5.69)	4.93 (4.42–5.31)	3–5	0.783
LDL (mmol/L)	2.86 (2.52–4.04)	2.83 (2.45–3.26)	-	0.326
HDL (mmol/L)	1.16 (0.99–1.31)	1.28 (1.12–1.71)	> 1	0.006
Triglycerides (mmol/L)	1.61 (1.00–2.75)	1.23 (0.95–1.65)	<1.7	0.049
TSH (μIU/mL)	1.53 (1.00–2.58)	1.98 (1.34–2.66)	0.27–4.20	0.322

All parameters are presented as the median and 1st–3rd quartile of the 25th–75th percentile. ALT—alanine transaminase; AST—aspartate transaminase; BMI—body mass index; GFR—glomerular filtration rate; HDL—high-density lipoprotein; LDL—low-density lipoprotein; TSH—thyroid-stimulating hormone.

### 4.3. Blood Platelet Isolation

All obtained blood samples were centrifuged immediately after transport (1200 rpm, 15 min, room temperature). Three-quarters of the platelet-rich plasma’s top layer was transferred to a fresh tube. To prepare pure blood platelet samples, we performed negative magnetic separation. Platelet-rich plasma was incubated with MicroBeads (Miltenyi Biotech, Bergisch Gladbach, Germany) conjugated with anti-CD45 (to avoid contamination by leukocytes) and anti-CD235a (to avoid contamination by erythrocytes). Positive separation with anti-CD61 was not recommended as a method for obtaining quiescent platelets. After cell labeling, MS columns were washed three times with 500 µL of modified wash buffer (PBS, 2 mM citrate, 0.5% BSA). Further, platelet-rich plasma was passed through the column. The labeled leukocytes and erythrocytes were retained in the column and pure platelets passed through to the new, fresh tube. After magnetic separation, purified platelet-rich plasma was centrifuged (1400 rpm, 15 min, room temperature) to obtain a blood platelet pellet. Isolated platelets were washed three times with modified Tyrode’s Buffer (127 mM NaCl, 2.7 mM KCl, 0.5 mM NaH_2_PO_4_, 12 mM NaHCO_3_, 5 mM HEPES, and 5.6 mM glucose, pH 7.4) to avoid contamination by plasma and suspended in the lysis buffer intended for 2D-DIGE (7 M urea, 2 M thiourea, 4% CHAPS, and 30 mM Tris) and in RNAlater (Invitrogen, Carlsbad, CA, USA) for the determination of mRNA expression levels. Prior to analysis, samples were stored at −80 °C.

### 4.4. Two-Dimensional Fluorescence Difference Gel Electrophoresis (2D-DIGE)

The 2D-DIGE was performed on the pooled samples from the study and control group. In the first stage, the protein concentration in the platelet samples was determined by the RCDC™ Protein Assay (BioRad Laboratories, Hercules, CA, USA). Samples were diluted to obtain 500 µg of proteins in the final volume of 100 µL and purified from ionic contaminants by ReadyPrep™ 2-D Cleanup Kit (BioRad Laboratories, Hercules, CA, USA). The protein concentration was determined one more time by RCDC™ Protein Assay after a clean-up procedure to prepare the appropriate dilution of samples required for fluorescent labeling by G-Dye200 and G-Dye300 markers from DyeAgnostics Refraction-2D QPLEX kit (NH DyeAGNOSTICS, Halle, Germany). After labeling, isoelectric focusing (IEF) of samples was performed on 11 cm IPG strips of a 3–10 pH gradient (BioRad Laboratories, Hercules, CA, USA) using PROTEAN i12 IEF Cell (BioRad Laboratories, Hercules, CA, USA). All IEF parameters were set according to the manufacturer’s protocol. Further, IPG strips were balanced in the Equilibration Buffer 1 (BioRad Laboratories, Hercules, CA, USA), containing dithiothreitol (DTT) for the reduction of proteins in the strip, and in Equilibration Buffer 2 (BioRad Laboratories, Hercules, CA, USA), containing iodoacetamide for alkylation of proteins in the strip. A second dimension separation was performed using Criterion™ TGX Precast Gels 4–20% (BioRad Laboratories, Hercules, CA, USA). All parameters for separation were set according to the manufacturer’s protocol. The fluorescently stained gel was scanned using a Typhoon FLA 950 GE scanner (GE Healthcare, Chicago, IL, USA) (G-Dye300 was used for samples from ACS patients, and G-Dye200 was used for control samples). Furthermore, a bioinformatics analysis of the obtained images was carried out using ImageMaster 2D Platinum 7.0 software (GE Healthcare, Chicago, IL, USA). Changes in protein expression were assessed based on a ratio of 1.5.

### 4.5. Nanoscale Liquid Chromatography Coupled to Tandem Mass Spectrometry (nanoLC-MS/MS)

Protein spots cut from a gel were decolorized with K_3_[Fe(Cn)_6_]/100 mM Na_2_SO_3_, rinsed with 100 mM NH_4_HCO_3_, shrunk with acetonitrile (AcN), and dried in a vacuum centrifuge. Next, samples were reduced with 10 mM DTT/100 mM NH_4_HCO_3_ for 45 min at 60 °C and alkylated in 55 mM iodoacetamide/100 mM NH_4_HCO_3_ for 30 min at room temperature in darkness. Proteins were digested overnight in a buffer containing 0.1 µg/µL trypsin at 37 °C with gentle shaking (150 rpm). Extraction of peptides from the gel was carried out in a solution of 50 mM NH_4_HCO_3_ and 2.5% TFA in 50% AcN by incubating the extracts in the ultrasonic bath. The extract was completely dried in a vacuum centrifuge and the samples were desalted using ZipTip C18 tips (Merck Millipore, Billerica, MA, USA). Peptides were suspended in a 0.1% TFA solution. Identification of digested proteins was performed using electrospray ionization tandem mass spectrometry (ESI-MS/MS) preceded by nanoLC-MS chromatographic separation with the application of the following conditions: gradient 4–99%; mass range 50–2000 m/z; composition of mobile phase: (A) 100% H_2_O; 0.05% TFA; (B) 80% AcN, 0.04% TFA. For high-resolution analysis, Acclaim™ PepMap™ and Acclaim PepMap RSLC™ columns were used (Trap column: Acclaim PepMap100 C18: particle size—5 µm; diameter—0.1 mm; length—20 mm and pore size—100 A; Analytic column Acclaim PepMap RSLC C18: particle size—2 um, diameter—0.05 mm, length—15 cm, pore size 100 A (Thermofisher, Waltham, MA, USA)). Proteins were identified on the MASCOT server (Swiss-Prot database) using ProteinScape software (Bruker, Billerica, MA, USA).

### 4.6. Determination of mRNA Expression Level of Identified Proteins in Platelets

The total RNA samples were isolated from purified blood platelets using the commercial kit ISOLATE II RNA Mini Kit (Bioline, London, UK). All steps were performed according to the manufacturer’s protocol. To avoid and remove genomic DNA contamination of samples, DNase I was used. The RNA samples were further transcribed into complementary DNA (cDNA) using Maxima First Strand cDNA Synthesis Kit (Thermofisher, Waltham, MA, USA) according to the attached instruction. The expression on the mRNA level was determined by Real-Time PCR Detection System Thermal Cycler CFX96TM (Bio Rad Laboratories Inc., Hercules, CA, USA) using TaqMan^®^ Gene Expression Assays and TaqMan^®^ Universal Master Mix, no. UNG. The following TaqMan^®^ Assays were used: 18S rRNA (Hs_99999901_s1), APOA1 (Hs_0098500_g1), VCL (Hs_00419715_m1), TAGLN2 (Hs_00761239_s1), TUBB1 (Hs_00917771_g1), FGB (Hs_00170586_m1), and FGG (Hs_00241037_m1). The relative expression levels of mRNA for all studied genes were calculated using −ΔCt.

### 4.7. Determination of Plasma Fibrinogen Concentration

The concentration of plasma fibrinogen in the study and control group was assessed by ELISA kit (Elabscience, Houston, TX, USA). Plasma samples were obtained after centrifugation of whole blood samples according to the manufacturer’s instructions. The concentration of fibrinogen was calculated by comparing the absorbance values of the samples to the standard curve.

### 4.8. Western Blot Analysis of Transgelin-2

Protein samples were first separated by the 12% sodium dodecyl sulfate-polyacrylamide gel electrophoresis (SDS-PAGE) (30 µg of protein mixture was applied onto the lane) and electrotransferred onto the Immobilon-P membrane (Millipore, Bedford, MA, USA). To assess the quality of the transfer, Ponceau S staining was performed. Obtained blots were incubated with the Anti-TAGLN2 antibody (Abcam, Cambridge, UK) for 2 h at room temperature with the 1:3000 dilution. Further, blots were washed with Tris-buffered saline buffer (TBS) containing 0.1% Tween-20 and incubated with the anti-rabbit IgG antibody (Santa Cruz Biotechnology, Dallas, TX, USA). Visualization of the proteins was performed on the X-ray film by the SuperSignal™ West Pico PLUS (Thermofisher, Waltham, MA, USA). Densitometric analysis of the protein bands on blots was carried out by Gel-Pro Analyzer Software 3.0 (Media Cybernetics Inc., Bethesda, MD, USA). To avoid any possible changes in the protein concentration, data were normalized by the IOD of total proteins after Ponceau S staining and also by the reference sample applied on all blots. As a reference sample, the protein mixture obtained from one patient was used and loaded onto each performed blot. The result of TAGLN2 is shown as a ratio of the IOD of the bands which corresponds to a relative protein level.

### 4.9. Statistical Analysis

All statistical analyses were performed in STATISTICA 13.3 software (StatSoft; Tulsa, OK, USA). The normality of distribution was assessed by the Shapiro–Wilk test. Depending on the Gaussian distribution, the Mann–Whitney U test or unpaired Student’s *t*-test was performed. A *p*-value < 0.05 was considered statistically significant. The range ratio was calculated as the ratio between the means or medians between studied groups.

## Figures and Tables

**Table 1:** List of identified proteins with altered expression in ACS patients in comparison to healthy donors by nanoscale liquid chromatography coupled to tandem mass spectrometry.

Spot Number	Protein	Molecular Weight (kDa)	pI	Ratio Range (RR)	Scores	# Peptides
S1	Vinculin	123.7	5.4	3.05223 (Downregulated)	767.1	16
S2	Fibrinogen β chain	55.9	9.3	1.72152 (Upregulated)	556.3	11
S3	Tubulin β chain	49.6	4.6	1.99851 (Upregulated)	202.4	12.2
S4	Apolipoprotein A1	30.8	5.5	2.38866 (Downregulated)	670.3	16
S5	Transgelin-2	22.4	9.3	2.12995 (Upregulated)	131.3	3
S6	Fibrinogen γ chain	51.5	5.3	1.94427 (Upregulated)	492.5	11

# Peptides—number of peptides.

## Data Availability

All data obtained from this study are included in the manuscript.
